# Vaccine Side Effects in Health Care Workers after Vaccination against SARS-CoV-2: Data from TüSeRe:exact Study

**DOI:** 10.3390/v15010065

**Published:** 2022-12-25

**Authors:** Alan Bareiß, Günalp Uzun, Marco Mikus, Matthias Becker, Karina Althaus, Nicole Schneiderhan-Marra, Axel Fürstberger, Julian D. Schwab, Hans A. Kestler, Martin Holderried, Peter Martus, Katja Schenke-Layland, Tamam Bakchoul

**Affiliations:** 1 Centre for Clinical Transfusion Medicine, 72076 Tübingen, Germany; 2 NMI Natural and Medical Sciences Institute, University Tübingen, 72770 Reutlingen, Germany; 3 Institute for Clinical and Experimental Transfusion Medicine, Medical Faculty of Tuebingen, University Hospital of Tübingen, 72076 Tübingen, Germany; 4 Institute of Medical Systems Biology, Ulm University, 89081 Ulm, Germany; 5 Department of Medical Structure, Process and Quality Management, University Hospital Tübingen, 72076 Tübingen, Germany; 6 Institute for Clinical Epidemiology and Applied Biostatistics, University Hospital Tübingen, 72076 Tübingen, Germany; 7 Institute of Biomedical Engineering, Department for Medical Technologies & Regenerative Medicine, Eberhard Karls University, 72076 Tübingen, Germany; 8 Cluster of Excellence iFIT (EXC 2180) “Image-Guided and Functionally Instructed Tumor Therapies”, Eberhard Karls University, 72076 Tübingen, Germany

**Keywords:** COVID-19, vaccine, reactogenicity, adverse event

## Abstract

As the Corona Disease 2019 (COVID-19) caused by SARS-CoV-2 persists, vaccination is one of the key measures to contain the spread. Side effects (SE) from vaccination are one of the reasons for reluctance to vaccinate. We systematically investigated self-reported SE after the first, second, and booster vaccinations. The data were collected during the TüSeRe: exact study (Tübinger Monitoring Studie zur exakten Analyse der Immunantwort nach Vakzinierung). Employees of health and research institutions were invited to participate. Study participants were asked to fill out an online questionnaire and report their SE after each dose of SARS-CoV-2 vaccination. A total of 1046 participants (mean age: 44 ± 12.9 years; female, *n* = 815 (78%); male, *n* = 231 (22%)) were included in the analysis. Local and systemic SE were more frequent after receiving the vector-based vaccine ChAdOx1 nCoV-19 in the first vaccination. However, local and systemic SE were more common after receiving mRNA vaccines (BNT162b2, mRNA-1273) in the second vaccination. Compared to the BNT162b2 vaccine, more SE have been observed after receiving the mRNA-1273 vaccine in the booster vaccination. In multivariate analysis, local and systemic side effects were associated with vaccine type, age and gender. Local and systemic SE are common after SARS-CoV-2 vaccines. The frequency of self-reported local and systemic SE differ significantly between mRNA and vector-based vaccines.

## 1. Introduction

Severe acute respiratory syndrome coronavirus 2 (SARS-CoV-2) is a novel strain of the long-known coronaviridae, which causes a complex of symptoms that are initially and predominantly respiratory in nature [1]. SARS-CoV-2 has been categorized as a positive sense single-strand RNA virus that is highly contagious. The SARS-CoV-2 was first identified in Wuhan, China, in 2019, forcing the World Health Organization (WHO) to declare an outbreak of crucial public health emergency and concern in January 2020 and a pandemic by March 2020 [2]. After its initial outbreak in Wuhan, WHO recommended the name 2019 novel coronavirus as a provisional name, however, by February, the international committee on taxonomy of virus officially recommended the name severe acute respiratory syndrome coronavirus 2 [3].

After identifying the first case of COVID-19 in China in 2019, the disease started to spread at a very high rate among humans in various parts of the globe, mainly through tiny droplets in the air that occur as a result of sneezing and coughing [4]. Some key symptoms that the COVID-19 patients recorded include fatigue, fever, loss of sense of smell, and dry cough.

The COVID-19 cases in Central Europe, France were first reported in January 2020, where three cases were detected, and the first death was also reported in the same location in February 2020 [5]. Later on, COVID-19 cases spread to other parts of Europe, causing numerous deaths that resulted in the creation and implementation of COVID-19 mitigation guidelines to contain the disease in Central Europe and other parts of the globe [6], as well as unprecedented efforts to understand the pathogenicity of SARS-CoV-2 [7].

There are numerous COVID-19 vaccines available and administered in various parts of the globe [8]. However, the vital COVID-19 vaccines available in Germany include, first, the vector-based ChAdOx-1 nCOV-19 vaccine (*Vaxzevria*, Oxford/AstraZeneca, UK) hereinafter referred to as *AZE*, which was recommended for people above 60 years of age and at high risk of contracting the disease because of its age-dependent safety profile [9]. Due to rare but severe thrombotic complications, this vaccine is no longer recommended [10]. Secondly, the BNT162b2 vaccine (Comirnaty, BioNTech/Pfizer, Mainz, Germany), hereinafter referred to as *BNT*, is administered to people 5 years of age and above. Both mRNA-1273 and BNT162b2 are mRNA-based vaccines. Thirdly, the mRNA-1273 (Spikevax, Moderna, Cambridge, MA, USA) vaccine, hereinafter referred to as *MOD*, which is considered effective and safe, particularly for people 30 years of age and above [11]. The vaccine is also be given to individuals who had previously contracted the coronavirus. For a complete immunization, two repeated doses of the same vaccine are necessary. However, due to vaccine availability, a combination of different vaccines has been also accepted as a full immunization. Since antibody titers are reduced with time, a booster vaccination 6 months after a full vaccination is deemed to be necessary for continued protection [12].

The COVID-19 vaccine administered triggers numerous side effects (SE) and reactions [13,14]. Acute side effects after a COVID-19 vaccine can be classified into two groups: local and systemic side effects. Local side effects include swelling, redness, pain on the injection site, and skin sensitivity. Systemic side effects include fatigue, diarrhea, nausea, muscle pain, joint pain, headache, shivering, and fever [15]. Most of the reported side effects usually diminish in a few days, however, on rare occasions, severe side effects such as anaphylaxis [16] and thrombotic events were reported [17,18].

Vaccine side effects are one of the reasons for reluctance to vaccinate [19]. In this study, we systematically investigated self-reported vaccine side effects after the first, second, and booster vaccinations.

## 2. Materials and Methods

The data were collected during the TüSeRe:exact Study (Tübinger Monitoring Studie zur exakten Analyse der Immunantwort nach Vakzinierung). TüSeRe:exact study aims to investigate the longitudinal changes in antibody levels after COVID-19 vaccines. Employees from the University Hospital Tübingen, the Center for Clinical Transfusion Medicine and Natural and Medical Sciences Institute Reutlingen were invited via e-mail to participate in the study.

Study participants were asked to fill out an online questionnaire and report side effects after receiving first, second, and booster vaccinations [20]. Regarding local side effects at the injection site, the probands were asked about were pain, skin sensitivity, swelling, and redness (i.e., erythema). The systemic side effects we asked about were headache, fever, shivers, generalized muscle pain, joint pain, fatigue, nausea, and diarrhea. These were acquired on an ordinal scale as follows: none, mild, moderate, severe.

Data are expressed as % (*n*) or as mean ± standard deviation (SD). The Mann–Whitney U test was used to test continuous variables. The frequency of adverse events was compared between vaccines using the Fisher’s exact test with Bonferroni correction. The severity of side effects was compared using the Kruskal–Wallis test with Dunn–Bonferroni correction between the different vaccines. A *p* < 0.05 is considered statistically significant. We used DATAtab: Online Statistics Calculator for statistical analysis (DATAtab e.U. Graz, Austria, https://datatab.net (accessed on 15 October 2022)). Multivariate analysis with a generalized linear model (GLM) and an adjustment for dependencies of observation between different vaccinations in the same subject (GEE, Generalized Estimating Equations) was performed using SPSS Version 29 (IBM Inc, Armonk, NY, USA). The presence of local and systemic side effects was included as a dependent parameter in the model. Variables were included in the models according to their statistical significance in univariate logistic regression analysis (*p* ≤ 0.1). Odds ratios (OR) with 95% confidence intervals (95%CI) were calculated.

## 3. Results

### 3.1. Study Cohort

A total of 1046 participants (mean age: 44 ± 12.9 years; female: *n* = 815 (78%), male: *n* = 231 (22%)) were included in the analysis. Overall, ChAdOx-1 nCOV-19 (AZE) accounted for 46.0% of the total administered first vaccines in participants, followed by BNT162b2 (BNT; 44%) and mRNA-1273 (MOD; 10%). Most of the participants received BNT as a second dose (in total 68.1%, *n* = 705), while 23% of the study sample population (*n* = 235) received MOD and 10% (*n* = 102) received AZE as second doses. Analysis shows that 74% of all participants (*n* = 772) received a third dose of vaccination, while 62% of the participants with a booster vaccination received BNT as the third vaccination, and the remaining (38%) received MOD as the third vaccine.

### 3.2. Local and Systemic Side Effects after First Vaccination

The percentage of participants reporting local or systemic side effects are presented in Table 1. After the first vaccination, 78% of the study cohort reported at least one local side effect. The most common side effect was pain at the injection site, which was reported by 73% of study participants.

The comparison of different vaccines in terms of side effect frequency is shown in Table 2. Local side effects were significantly different between AZE and BNT only in terms of skin sensitivity (Table 3). Swelling and erythema were significantly higher after MOD compared to AZE (*p* < 0.05, Table 3). Furthermore, skin sensitivity, swelling, and redness were more frequent after MOD compared to BNT (*p* < 0.05, Table 2).

**Figure 1:**
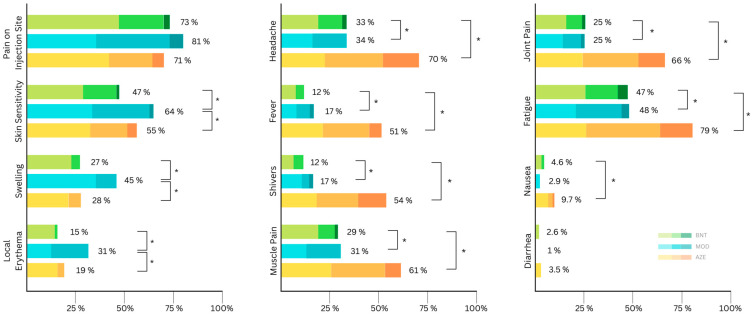
Distribution of self-reported side effects according to symptom severity (mild, moderate, severe) after the first vaccination (AZE:AstraZeneca (ChAdOx1 nCov19); BNT: Biontech/Pfizer (BNT162b2); MOD: Moderna (mRNA-1273)). Asterisks indicate significant differences in severity.

The distribution of local side effects according to symptom severity is presented in Figure 1. Compared to AZE, the severity of local side effects, except for skin sensitivity and diarrhea, was significantly higher after vaccination with MOD (Table 3). However, the severity of most local side effects was similar after receiving AZE and BNT vaccines after the first vaccination (Table 3). On the other hand, the severity of most of the local side effects was higher after MOD compared to BNT (Table 3).

At least one systemic side effect was reported by 72% of the study participants (Table 2). The most frequent side effect was fatigue, which was reported by 62% of the participants. Systemic side effects were reported by 88% of those participants receiving AZE as the first vaccine. In contrast, the percentage of participants with systemic side effects was 60% and 57% after receiving BNT and MOD vaccines, respectively. All systemic side effects, except nausea and diarrhea, differed significantly between AZE and both mRNA-based vaccines (Table 2). The severity and frequency of self-reported systemic side effects are presented in Figure 1. In terms of the severity of systemic side effects, BNT and MOD vaccines were not significantly different (Table 3).

### 3.3. Local and Systemic Side Effects after the Second Vaccination

**Figure 2:**
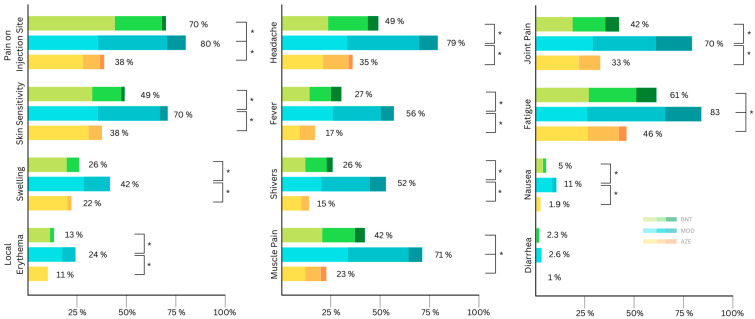
Distribution of self-reported local side effects according to symptom severity (mild, moderate, severe) after the second vaccination (AZE: AstraZeneca (ChAdOx1 nCov19); BNT: Biontech/Pfizer (BNT162b2); MOD: Moderna (mRNA-1273)). Asterisks indicate significant differences in severity.

The overall percentage of study participants that reported any local and systemic side effects were similar after the second vaccination, being 75% and 73% (Table 4), respectively. The most reported local side effect was pain at the injection site, reported by 87%, and the most common systemic side effect was fatigue, with 64%. Recipients of the MOD vaccine as the second dose not only reported the highest proportion of local side effects, but also systemic ones, where the difference is most significant (Table 4). All local side effects were more frequent after MOD compared to AZE and BNT (Table 4). Pain at the injection site was more common after BNT compared to AZE (Table 4). Although the frequency of skin sensitivity was similar after receiving AZE and BNT, the severity of the symptom was significantly higher after receiving BNT (Figure 2 and Table 3).

All side effects apart from diarrhea were reported with a significantly (statistically) higher frequency and severity after receiving MOD compared to after receiving AZE or BNT (Table 3 and Table 4). AZE had the lowest proportion of reported adverse events compared with the first dose, with 53% of the participants experiencing local side effects and 54% experiencing systemic side effects (Table 4). General muscle pain and fatigue were significantly more common after BNT compared to AZE (Table 4). The vast majority of all reported side effects after the second dose were mild to moderate (Figure 2).

### 3.4. Local and Systemic Side Effects after Booster Vaccination

**Figure 3:**
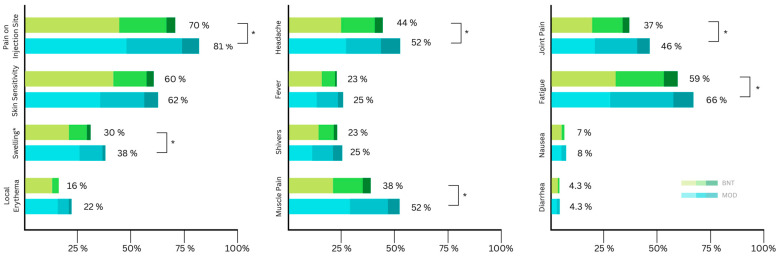
Distribution of self-reported local side effects according to symptom severity (mild, moderate, severe) after the third (booster) vaccination (BNT: Biontech/Pfizer (BNT162b2); MOD: Moderna (mRNA-1273)). Asterisks indicate significant differences in severity.

A total of 772 participants received a booster vaccine. Of these, 720 participants filled out the online questionnaires about side effects. In accordance with current local recommendations, no participants received AZE as a third dose (Table 5). The proportion of recipients reporting at least one side effect locally or systemically is comparable to the previous two administrations. Again, pain at the injection site (75%) and fatigue (62%) were the highest reported side effects (Figure 3). A statistically significant difference in frequency between MOD and BNT was found in seven of the twelve included side effects (Table 5) and five out of the twelve in terms of severity (Table 3).

### 3.5. Homologous vs. Heterologous Vaccine Regimes

We also investigated the side effect frequency after homologous and heterologous vaccine regimes. The heterologous vaccine regime with AZE in the first vaccination and BNT in the second vaccination resulted in an increased frequency of pain at the injection site and general muscle pain after the second vaccination compared to the heterologous vaccine regime with AZE (Table 6). However, a statistically significant difference between AZE-MOD and AZE-AZE vaccine regimes was found in two of four local side effects and six of twelve side effects (Table 6). On the other hand, the incidence of side effects was similar when the second vaccination was performed with the same mRNA vaccine.

### 3.6. Multivariate Analysis

We analyzed the associations between vaccine type and any local or systemic SE in all participants (Table 7). As risk factors for local SEs, our model took vaccine type, age, skin disease, and previous COVID-19 infection into consideration. Multivariate analyses showed an association between vaccine type and the frequency of local SE (Table 7). In terms of local SE, MOD showed higher odds ratios for local SE compared to both AZE (OR(95%CI) = 2.202 (1.630–2.975); *p* < 0.001) and BNT (OR(95%CI) = 1.799 (1.344–2.408); *p* = 0.171). While the difference between BNT and AZE was not significant (OR(95%CI) = 1.224 (0.966–1.551); *p* = 0.094). Younger age (18–45 years) was associated with higher odds ratio for local SE (OR(95%CI)= 1.817 (1.431–2.332); *p* < 0.001). Having a skin disease was associated with higher odds ratio of having ≥1 local SE (OR(95%CI) = 2.914 (1.390–6.109); *p* = 0.005). In contrast, previous COVID-19 infection was not associated with increased local SE (OR(95%CI) = 0.667 (0.373–1.192); *p* = 0.171).

In terms of systemic SEs, vaccine type, age, skin disease, and cardiovascular disease were analyzed in the model (Table 7). Similar to local SEs, vaccine type was associated with the frequency of having ≥1 systemic SE. The difference between MOD and AZE was not significant (OR(95%CI) = 0.785 (0.596–1.034); *p* = 0.085). On the other hand, BNT was associated with lower SE frequency compared to both MOD (*p* < 0.001) and AZE (*p* < 0.001) vaccines. The frequency of systemic SE was higher in participants younger than 45 years of age (OR(95%CI) = 1.458 (1.177–1.805); *p* < 0.001). Cardiovascular disease was associated with higher incidence of systemic SE (OR(95%CI) = 1.575 (1.007–2.463); *p* = 0.046).

Although the differences between vaccines remained significant after adjustment, we found larger differences between the effects of vaccines in younger patients compared to older ones for local SE, but not for systemic SE (Supplemental Tables S1 and S2). The same held true for males (larger differences between vaccines) vs. females in local SE (Supplemental Tables S3 and S4).

## 4. Discussion

Dominating not only the scientific efforts of the last two and a half years globally but also the mainstream media, the topic of COVID-19 has caused an unprecedented social division in ethical, medical, scientific, and socio-political standpoints. Misinformation has reached a new peak. By continuing the effort of monitoring both the subjective position of the population receiving vaccines as well as collecting quantifiable and absolute data on the immune response, we are able to enhance trust in scientific research, can prove the effectiveness of vaccines, or raise concern by monitoring vaccine breakthroughs. As of today, the COVID-19 pandemic is continuing, thus the scientific effort around the multi-faceted subject matter of COVID-19 should continue.

In the current study, we investigated self-reported acute side effects after first, second and third vaccinations. After the first vaccination, although local side effects were more common after mRNA vaccines, systemic side effects were more common and severe after AZE. Similarly, Briggs et al. reported a higher side effect rate after receiving AZE compared to BNT after the first dose [21]. Furthermore, Klugar et al. compared mRNA-based and viral vector based COVID-19 vaccines among healthcare workers after receiving the first vaccination [22]. Similar to our findings, while local side effects were more common after receiving mRNA-based vaccines compared to viral vector-based vaccines (78.3% vs 70.4%), systemic side effects were more frequent after viral vector-based vaccines (62% vs 87%) [22]. The fact that a vector vaccine carries both the antigen and the viral vector, and both can elicit an immune response, it may explain the higher reactogenicity of vector vaccines [23]. Furthermore, high prevalence of adenoviral diseases in the community and the consecutive pre-existing immune mechanisms might also contribute to these findings [24,25].

In further analysis of our data, we observed that local side effects were more common after MOD compared to BNT, however, only some systemic symptoms were significantly different between BNT and MOD. Previous studies reported a higher side effect rate after MOD compared to BNT [26]. Both vaccines are mRNA-based and are packed in lipid particles and contain no adjuvants. However, potentially explaining the difference, is the dose per injection, which is significantly higher in MOD (100 µg) compared to BNT (30 µg).

After the second dose, the frequency of side effects reduced if AZE was administered. Kaur et al. investigated local and systemic side effects after vaccination with AZE in health care workers [13]. Similar to our findings, they found that side effects are more common after the first dose compared to the second dose [13]. This effect may be in part explained by the adjustment of the German vaccination recommendations. AZE recipients were significantly older. As Ramasamy et al. have shown, the tolerance of AZE is higher in older adults [27]. On the other hand, we found that compared to the first dose, the number of individuals reporting systemic side effects increased among those receiving an mRNA vaccine as a second dose. This finding is in accordance with the literature. El-Shitany et al. also reported an increased side effect rate after a second dose of BNT compared to the first vaccine dose [15]. It can be explained through a two-fold mechanism: the build-up of long-lasting memory T-cells and B-cells facilitates a faster and more intense immune response. And as discussed by Yao et al., a more recent finding suggests an additional role of the cells of the innate immune system in contributing to a well-prepared immune response upon a second encounter with an immunogen. This phenomenon is referred to as trained immunity [28].

For primary vaccination, a second vaccine dose with the same vaccine is recommended. However, due to limited vaccine availability, heterologous vaccine regimes were also approved by regulatory agencies [29]. Furthermore, heterologous vaccination provides a better immunogenicity compared to homologous vaccination [30]. We further investigated whether heterologous vaccination influenced the side effect frequency. Both local and systemic side effects were more common in individuals receiving MOD or BNT after AZE compared to those who received two doses of AZE. Hillus et al. recently found systemic reactions in 49% of vaccinations after heterologous AZE–BNT vaccination, and in 39% after homologous AZE–AZE [31]. Baldolli et al. investigated the side effect rate after heterologous vaccination in health care workers [32]. In accordance with our findings, individuals receiving the mRNA-1273 vaccine reported more local and systemic symptoms compared with those receiving the BNT162b2 vaccine after AZE [32]. 

Due to declining antibody titers, booster vaccination was recommended to prolong protection against COVID-19. Furthermore, it has been shown that the antibodies that are developed after vaccines using the spike protein of a wild-type virus have a limited neutralization ability against new variants of the virus [33]. A third vaccination improves the antibody binding to the omicron variant [33]. Most of the participants in our study received BNT as a booster. Local side effects were more common after MOD compared to BNT in booster vaccination.

Age and gender might affect the reactogenicity to COVID-19 vaccination [14,15,34]. Using multivariate analyses, we investigated possible confounders. Differences between vaccines remained significant after adjustment. In agreement with previous studies [15,35], multivariate analyses in our study revealed a tendency to local and systemic SE in participants older than 45 years of age. Furthermore, females reported more side effects compared to males in our cohort. These results indicated that age and sex were associated with vaccine side effects and that these effects should be considered when interpreting the results of similar studies investigating reactogenicity after COVID-19 vaccination.

We also found that people with skin diseases had a higher odds ratio for local SE but not for systemic SE. Similarly, cardiovascular diseases were associated with higher systemic SE. These findings might be important in pre-vaccination information. However, further studies are needed to better understand this phenomenon. However, these results should be interpreted cautiously because the number of persons with investigated comorbidities was small.

Prior COVID-19 infection might be associated with more frequent and severe SE after COVID-19 vaccination [35]. However, in the current study, previous COVID-19 infection was not found to be associated with SE. We believe the low number of participants with prior COVID-19 infection (3%) precludes any certain association.

The strengths of our study are the collection of real-world data in a special population after different vaccine schemes and high study adherence after the 1st, 2nd, and booster vaccinations.

Still, our study has limitations. First, the number of participants was relatively small compared to other population-based side effect studies supported by government agencies. However, this study targeted a specific occupational group and included around 1000 participants. In addition, the continuous follow-up of study participants allowed the collection of data on adverse events after the 1st, 2nd, and 3rd vaccinations, and to study the effect of different vaccination regimens. Second, the study population consisted mainly of women. In Germany, approximately 75% of health care workers are female [36]. Therefore, the gender distribution reflects the population under study. Third, side effects were collected using a pre-formed online questionnaire, which might cause the underreporting of rare side effects. Online questionnaires and applications have recently been increasingly used to collect information for medical studies when face-to-face interviews are not possible because of contact limitations. On the other hand, the studied population consisted of health professionals who can be assumed to have the necessary health literacy to understand and answer the health-related questions.

## 5. Conclusions

In conclusion, local and systemic acute side effects are frequently reported after COVID-19 vaccines. The rate of side effects declines after a second dose of AZE but increases after receiving BNT and MOD. Furthermore, SE were reported more frequently after the second dose when an mRNA vaccine was administered subsequent to a vector-based vaccine compared to two homologous doses of a vector vaccine. Also, we found that the frequency of side effects changes after booster vaccination. In multivariate analysis, local and systemic SE were associated with vaccine type, gender, and age. Addressing this in the pre-vaccination patient information should be considered by physicians. The connection of reactogenicity to immunogenicity needs further scientific attention.

## Figures and Tables

**Table 1:** General cohort characteristics.

***n***	1046	
**Gender**		
male	231 (22%)	
female	814 (78%)	
Age	44.35 ± 12.89	
Comorbidites	Frequency	% of Cases
Cardiovascular	71	7%
Pulmonologic	38	4%
Tumor	34	3%
Gastrointestinal	29	3%
Skin	49	5%
Neurologic	20	2%
Hematogenic	13	1%
Hepatic / Nephrologic	10	1%
Any Chronic Condition	173	17%
Infection before V1	30	3%
Infection before V2	38	4%
Infection before V3	25	2%

**Table 2:** Acute local and systemic side effects after the first vaccination.

	Total (*n*)	BNT	MOD	AZE			
*n*	1046	460	103	483			
Age (± SD)	44 (±12.9)	43 (±13)	40 (±14)	46 (±12)			
Gender, % (*n*)							
male	22 (231)	25 (114)	24 (25)	19 (92)	**BNT vs.**	**BNT vs.**	**MOD vs.**
female	78 (815)	75 (346)	74 (78)	81 (391)	**AZE**	**MOD**	**AZE**
**local side effects, % (*n*)**	78 (821)	79 (362)	86 (89)	77 (370)	freq.	freq.	freq.
pain on injection site	73 (762)	73 (338)	80 (83)	71 (341)	1	0.798	0.24
skin sensitivity	52 (547)	47 (215)	64 (66)	55 (266)	0.06	0.006 *	0.564
swelling	29 (307)	27 (124)	46 (47)	28 (136)	1	0.006 *	0.006 *
local erythema	19 (195)	15 (71)	31 (195)	19 (92)	0.852	0.006 *	0.042 *
**systemic side effects** **% (*n*)**	72 (761)	60 (277)	57 (59)	88 (425)			
headache	50 (527)	33 (154)	34 (35)	70 (338)	0.006 *	1	0.006 *
fever	30 (319)	12 (54)	17 (17)	51 (248)	0.006 *	1	0.006 *
shivers	32 (331)	12 (53)	17 (17)	54 (261)	0.006 *	0.996	0.006 *
general muscle pain	44 (457)	29 (132)	31 (32)	61 (293)	0.006 *	1	0.006 *
joint pain	44 (461)	25 (116)	25 (26)	66 (319)	0.006 *	1	0.006 *
fatigue	62 (646)	47 (215)	48 (49)	79 (382)	0.006 *	1	0.006 *
nausea	7 (71)	5 (21)	3 (3)	10 (47)	0.012 *	1	0.15
diarrhea	3 (30)	3 (12)	1 (1)	4 (17)	1	1	1

*p*-values for the comparison of different vaccines in terms of side effect frequency and severity. A Chi-square test has been performed for the administered vaccine on first vaccination and the proportion of each reported side effect. *p*-values were adjusted with the Bonferroni correction (6×). Asterisks indicate statistical significance. (AZE: AstraZeneca (ChAdOx1 nCov19); BNT: Biontech/Pfizer (BNT162b2); MOD: Moderna (mRNA-1273)).

**Table 3:** Comparison of side effects in terms of symptom severity after different vaccines.

	AZE vs. BNT		AZE vs. MOD		BNT vs. MOD		
	V1	V2	V1	V2	V1	V2	V3
**local side effects**							
pain on injection site	1	00.001 *	00.01 *	00.001 *	00.005	00.001 *	00.001 *
skin sensitivity	00.037 *	00.116	00.094	00.001 *	00.001 *	00.001 *	00.131
swelling	1	00.918	00.03 *	00.001 *	00.006 *	00.001 *	00.111
redness	00.586	1	00.035	00.32	00.003 *	00.001 *	00.22
** systemic side effects **							
headache	00.001 *	00.1	00.001 *	00.001 *	1	00.001 *	00.029 *
fever	00.001 *	00.164	0.001 *	0.001 *	0.618	0.001 *	0.438
shivers	0.001 *	0.076	0.001 *	0.001 *	0.481	0.001 *	0.39
general muscle pain	0.001 *	0.035 *	0.001 *	0.001 *	0.664	0.001 *	0.003 *
joint pain	0.001 *	0.419	0.001 *	0.001 *	1	0.001 *	0.015 *
fatigue	0.001 *	0.019 *	0.001 *	0.001 *	1	0.001 *	0.012 *
nausea	0.07 *	1	0.105	0.005 *	1	0.001 *	0.892
diarrhea	1	1	0.222	0.877	0.654	0.877	0.989

(AZE: AstraZeneca (ChAdOx1 nCov19); BNT: Biontech/Pfizer (BNT162b2); MOD: Moderna (mRNA-1273)). The table shows *p*-values. For first (V1) and second vaccines (V2), the *p* values were adjusted with the Bonferroni correction. Asterisks indicate statistical significance.

**Table 4:** Acute local and systemic side effects after the second vaccination. *p*-values for the comparison of different vaccines in terms of side effect frequency and severity. A Chi-square test was performed for the administered vaccine after the first vaccination and the proportion of each reported side effect. *p*-values were adjusted with the Bonferroni correction (6×). Asterisks indicate statistical significance. (AZE: AstraZeneca (ChAdOx1 nCov19); BNT: Biontech/Pfizer (BNT162b2); MOD: Moderna (mRNA-1273)).

	Total (*n*)	BNT	MOD	AZE			
* n *	1042	705	235	102			
Age (± SD)	43 (±13)	44 (±12)	42 (±12)	54 (±10)			
Gender, % (*n*)							
male	22 (231)	23 (164)	19 (46)	21 (21)	**BNT vs.**	**BNT vs.**	**MOD vs.**
female	78 (815)	77 (542)	81 (192)	79 (81)	**AZE**	**MOD**	**AZE**
**local side effects, % (*n*)**	75 (782)	75 (528)	85 (200)	53 (54)	freq.	freq.	freq.
pain on injection site	87 (718)	70 (492)	80 (187)	38 (39)	0.006 *	0.018 *	0.018 *
skin sensitivity	52 (546)	49 (342)	70 (165)	38 (39)	0.33	0.006 *	0.006 *
swelling	29 (303)	26 (182)	42 (99)	22 (22)	1	0.006 *	0.024 *
local erythema	16 (163)	13 (95)	24 (57)	11 (11)	1	0.006 *	0.03 *
** systemic side effects ** **% (*n*)**	73 (771)	71 (497)	92 (216)	57 (58)			
headache	54 (563)	49 (342)	79 (185)	35 (36)	0.072	0.006 *	0.006 *
fever	32 (338)	27 (189)	56 (132)	17 (17)	0.174	0.006 *	0.006 *
shivers	30 (317)	26 (180)	52 (122)	15 (15)	0.108	0.006 *	0.006 *
general muscle pain	46 (482)	42 (293)	71 (166)	23 (23)	0.006 *	0.006 *	0.006 *
joint pain	47 (493)	42 (294)	70 (165)	33 (34)	0.678	0.006 *	0.006 *
fatigue	64 (670)	61 (427)	83 (196)	46 (47)	0.036	0.006 *	0.006 *
nausea	6 (65)	5 (37)	11 (26)	2 (2)	0.894	0.012 *	0.03 *
diarrhea	2 (23)	2 (16)	3 (6)	1 (1)	1	1	1

**Table 5:** Acute local and systemic side effects after the third (booster) vaccination. Asterisks indicate statistical significance. (BNT: Biontech/Pfizer (BNT162b2); MOD: Moderna (mRNA-1273)). *p*-values indicate the significance level of the difference in frequency.

	Total (*n*)	BNT	MOD	
* n *	720	474	295	
Age (± SD)	43 (±13)	42 (±13)	49 (±10)	
Gender, % (*n*)				
male	21 (205)	23 (107)	21 (61)	**BNT vs.**
female	79 (761)	77 (366)	79 (232)	**MOD**
**local side effects, % (*n*)**	80 (579)	78 (344)	85 (235)	frequency
pain on injection site	75 (537)	70 (312)	81 (225)	0.001 *
skin sensitivity	61 (439)	61 (267)	62 (172)	0.626
swelling	33 (241)	31 (136)	38 (105)	0.046 *
local erythema	18 (133)	16 (73)	22 (60)	0.081
** systemic side effects ** **% (*n*)**	70 (502)	66 (292)	86 (210)	
headache	47 (339)	44 (195)	52 (144)	0.037 *
fever	24 (171)	23 (101)	25 (70)	0.488
shivers	24 (170)	23 (100)	25 (70)	0.407
general muscle pain	43 (313)	38 (170)	52 (143)	0.001 *
joint pain	40 (289)	37 (162)	46 (127)	0.013 *
fatigue	62 (446)	59 (262)	66 (184)	0.05 *
nausea	7 (52)	7 (31)	7 (21)	0.769
diarrhea	4 (31)	4 (19)	4 (12)	0.978

**Table 6:** Comparison of side effects in terms of symptom frequency after different vaccination schemes (AZE:AstraZeneca (ChAdOx1 nCov19); BNT: Biontech/Pfizer (BNT162b2); MOD: Moderna (mRNA-1273)). The table shows the percentage of vaccinees that reported any local or systemic SE, as well as each symptom, respectively, and the corresponding *p*-values of inter-scheme analysis after the Bonferroni correction (20×). Asterisks indicate statistical significance.

	AZE-AZE	AZE-BNT	AZE-MOD	BNT-BNT	MOD-MOD	BNT-BNT vs. AZE-BNT	MOD-MOD vs. AZE-MOD	AZE-AZE vs. AZE-BNT	AZE-AZE vs. AZE-MOD
* n *	102	249	133	460	102				
local side effects, % (*n*)	53 (54)	70 (174)	82 (109)	77 (354)	89 (91)				
pain on injection site	39 (39)	66 (163)	67 (102)	72 (329)	83 (85)	1	1	0.02 *	0.02 *
skin sensitivity	39 (39)	50 (125)	69 (92)	47 (217)	72 (73)	0.98	1	0.9	0.02 *
swelling	22 (22)	25 (63)	38 (50)	26 (119)	48 (49)	1	1	1	0.18
local erythema	11 (11)	14 (35)	18 (24)	13 (60)	32 (33)	1	0.22	1	1
systemic side effects % (*n*)	57 (58)	70 (174)	90 (120)	71 (323)	94 (96)				
headache	36 (36)	50 (125)	77 (102)	47 (217)	80 (83)	1	1	0.24	0.02 *
fever	17 (17)	31 (78)	47 (63)	24 (111)	68 (69)	0.8	0.04 *	0.1	0.02 *
shivers	15 (15)	27 (67)	48 (64)	25 (113)	57 (58)	1	1	0.3	0.02 *
general muscle pain	23 (23)	40 (100)	71 (95)	42 (193)	70 (71)	1	1	0.04 *	0.02 *
joint pain	34 (34)	38 (95)	72 (96)	44 (199)	68 (69)	1	1	1	0.02 *
fatigue	47 (47)	62 (153)	82 (109)	60 (274)	85 (87)	1	1	0.18	0.02 *
nausea	2 (2)	4 (10)	8 (11)	6 (27)	14 (15)	1	1	1	0.74
diarrhea	1 (1)	3 (7)	3 (4)	2 (9)	2 (2)	1	1	1	1

**Table 7:** Generalized linear model across all vaccination instances (V1, V2 and V3) for local and systemic SE.

				95% Confidence Interval	
Side Effects	Parameter	*p*	Odds Ratio	Lower	Upper
Local	BNT vs. AZE	0.094	1.224	0.966	1.551
	MOD vs. AZE	<0.001	2.202	1.630	2.975
	MOD vs. BNT	<0.001	1.799	1.344	2.408
	females vs. males	<0.001	1.806	1.441	2.263
	Age 18-45 vs. Age > 45	<0.001	1.827	1.431	2.332
	Skin disease	0.005	2.914	1.390	6.109
	Previous COVID-19	0.171	0.667	0.373	1.192
Systemic	BNT vs. AZE	<0.001	0.404	0.316	0.515
	MOD vs. AZE	0.085	0.785	0.596	1.034
	MOD vs. BNT	<0.001	1.945	1.552	2.438
	females vs. males	<0.001	1.718	1.388	2.127
	Age 18-45 vs. Age > 45	<0.001	1.458	1.177	1.805
	Skin disease	0.071	1.585	0.962	2.612
	Previous COVID-19	0.046	1.575	1.007	2.463

## Data Availability

Data may be requested for academic collaboration from the corresponding author.

## Supplementary Materials

The following supporting information can be downloaded at: https://www.mdpi.com/article/10.3390/v15010065/s1, Table S1: Generalized linear model across all vaccination instances (i.e. V1, V2 and V3) for local SE according to age groups; Table S2: Generalized linear model across all vaccination instances (i.e. V1, V2 and V3) for local SE according to age groups; Table S3: Generalized linear model across all vaccination instances (i.e. V1, V2 and V3) for local SE in males and females; Table S4: Generalized linear model across all vaccination instances (i.e. V1, V2 and V3) for systemic SE in males and females.

## References

[1] Chung J.Y., Thone M.N., Kwon Y.J. (2021). COVID-19 vaccines: The status and perspectives in delivery points of view. Adv. Drug Deliv. Rev..

[2] Chaw S.-M., Tai J.-H., Chen S.-L., Hsieh C.-H., Chang S.-Y., Yeh S.-H., Yang W.-S., Chen P.-J., Wang H.-Y. (2020). The origin and underlying driving forces of the SARS-CoV-2 outbreak. J. Biomed. Sci..

[3] Abrignani M.G., Murrone A., de Luca L., Roncon L., Di Lenarda A., Valente S., Caldarola P., Riccio C., Oliva F., Gulizia M.M. (2022). COVID-19, Vaccines, and Thrombotic Events: A Narrative Review. J. Clin. Med..

[4] Singh D., Yi S.V. (2021). On the origin and evolution of SARS-CoV-2. Exp. Mol. Med..

[5] Spiteri G., Fielding J., Diercke M., Campese C., Enouf V., Gaymard A., Bella A., Sognamiglio P., Sierra Moros M.J., Riutort A.N. (2020). First cases of coronavirus disease 2019 (COVID-19) in the WHO European Region, 24 January to 21 February 2020. Eurosurveillance.

[6] Nicola M., O’Neill N., Sohrabi C., Khan M., Agha M., Agha R. (2020). Evidence based management guideline for the COVID-19 pandemic—Review article. Int. J. Surg..

[7] Wang M.-Y., Zhao R., Gao L.-J., Gao X.-F., Wang D.-P., Cao J.-M. (2020). SARS-CoV-2: Structure, Biology, and Structure-Based Therapeutics Development. Front. Cell. Infect. Microbiol..

[8] R&D Blueprint COVID-19 Vaccine Tracker and Landscape. https://www.who.int/publications/m/item/draft-landscape-of-covid-19-candidate-vaccines.

[9] Riad A., Pokorná A., Mekhemar M., Conrad J., Klugarová J., Koščík M., Klugar M., Attia S. (2021). Safety of ChAdOx1 nCoV-19 Vaccine: Independent Evidence from Two EU States. Vaccines.

[10] Robert Koch-Institut Epidemiologisches Bulletin 21/2022. www.rki.de/epidbull.

[11] Leav B., Straus W., White P., Leav A., Gaines T., Maggiacomo G., Kim D., Smith E.R., Gurwith M., Chen R.T. (2022). A Brighton Collaboration standardized template with key considerations for a benefit/risk assessment for the Moderna COVID-19 Vaccine (mRNA-1273). Vaccine.

[12] Bar-On Y.M., Goldberg Y., Mandel M., Bodenheimer O., Freedman L., Kalkstein N., Mizrahi B., Alroy-Preis S., Ash N., Milo R. (2021). Protection of BNT162b2 Vaccine Booster against Covid-19 in Israel. N. Engl. J. Med..

[13] Kaur S., Singh A., Saini S., Rohilla L., Kaur J., Chandi A., Kaur G., Singh M., Kumar P., Soni S.L. (2022). Reporting adverse events of ChAdOx1 nCoV-19 coronavirus vaccine (Recombinant) among the vaccinated healthcare professionals: A cross-sectional survey. Indian J. Med. Res..

[14] Oueijan R.I., Hill O.R., Ahiawodzi P.D., Fasinu P.S., Thompson D.K. (2022). Rare Heterogeneous Adverse Events Associated with mRNA-Based COVID-19 Vaccines: A Systematic Review. Medicines.

[15] El-Shitany N.A., Harakeh S., Badr-Eldin S.M., Bagher A.M., Eid B., Almukadi H., Alghamdi B.S., Alahmadi A.A., Hassan N.A., Sindi N. (2021). Minor to Moderate Side Effects of Pfizer-BioNTech COVID-19 Vaccine Among Saudi Residents: A Retrospective Cross-Sectional Study. Int. J. Gen. Med..

[16] Castells M.C., Phillips E.J. (2021). Maintaining Safety with SARS-CoV-2 Vaccines. N. Engl. J. Med..

[17] Schulz J.B., Berlit P., Diener H.-C., Gerloff C., Greinacher A., Klein C., Petzold G.C., Piccininni M., Poli S., Röhrig R. (2021). COVID-19 Vaccine-Associated Cerebral Venous Thrombosis in Germany. Ann. Neurol..

[18] Althaus K., Möller P., Uzun G., Singh A., Beck A., Bettag M., Bösmüller H., Guthoff M., Dorn F., Petzold G.C. (2021). Antibody-mediated procoagulant platelets in SARS-CoV-2-vaccination associated immune thrombotic thrombocytopenia. Haematologica.

[19] Mongua-Rodríguez N., Rodríguez-Álvarez M., De-la-Rosa-Zamboni D., Jiménez-Corona M.E., Castañeda-Cediel M.L., Miranda-Novales G., Cruz-Pacheco G., Ferreira-Guerrero E., Ferreyra-Reyes L., Delgado-Sánchez G. (2022). Knowledge, attitudes, perceptions, and COVID-19 hesitancy in a large public university in Mexico city during the early vaccination rollout. BMC Public Health.

[20] Völkel G., Fürstberger A., Schwab J.D., Werle S.D., Ikonomi N., Gscheidmeier T., Kraus J.M., Groß A., Holderried M., Balig J. (2021). Patient Empowerment During the COVID-19 Pandemic by Ensuring Safe and Fast Communication of Test Results: Implementation and Performance of a Tracking System. J. Med. Internet Res..

[21] Briggs F.B.S., Mateen F.J., Schmidt H., Currie K.M., Siefers H.M., Crouthamel S., Bebo B.F., Fiol J., Racke M.K., O’Connor K.C. (2022). COVID-19 Vaccination Reactogenicity in Persons With Multiple Sclerosis. Neurol. Neuroimmunol. Neuroinflamm..

[22] Klugar M., Riad A., Mekhemar M., Conrad J., Buchbender M., Howaldt H.-P., Attia S. (2021). Side Effects of mRNA-Based and Viral Vector-Based COVID-19 Vaccines among German Healthcare Workers. Biology.

[23] Morales-Núñez J.J., Muñoz-Valle J.F., Machado-Sulbarán A.C., Díaz-Pérez S.A., Torres-Hernández P.C., Panduro-Espinoza B.V., Gallegos-Díaz de Leon J.A., Munguía-Ramirez C.D., Hernández-Bello J. (2022). Comparison of three different COVID-19 vaccine platforms (CoronaVac, BTN162b2, and Ad5-nCoV) in individuals with and without prior COVID-19: Reactogenicity and neutralizing antibodies. Immunol. Lett..

[24] Atasheva S., Shayakhmetov D.M. (2016). Adenovirus sensing by the immune system. Curr. Opin. Virol..

[25] Lynch J.P., Kajon A.E. (2016). Adenovirus: Epidemiology, Global Spread of Novel Serotypes, and Advances in Treatment and Prevention. Semin. Respir. Crit. Care Med..

[26] Sáez-Peñataro J., Torres F., Bartra J., Bascuas J., Vilella A., Tortajada M., Quesada S., González E., López-Suñé E., Castells A. (2022). Tolerability and Reactogenicity Profile of mRNA SARS-Cov-2 Vaccines from a Mass Vaccination Campaign in a Tertiary Hospital: Between-Vaccine and Between-Population Prospective Observational Study (VigilVacCOVID Study). BioDrugs.

[27] Ramasamy M.N., Minassian A.M., Ewer K.J., Flaxman A.L., Folegatti P.M., Owens D.R., Voysey M., Aley P.K., Angus B., Babbage G. (2021). Safety and immunogenicity of ChAdOx1 nCoV-19 vaccine administered in a prime-boost regimen in young and old adults (COV002): A single-blind, randomised, controlled, phase 2/3 trial. Lancet.

[28] Yao Y., Jeyanathan M., Haddadi S., Barra N.G., Vaseghi-Shanjani M., Damjanovic D., Lai R., Afkhami S., Chen Y., Dvorkin-Gheva A. (2018). Induction of Autonomous Memory Alveolar Macrophages Requires T Cell Help and Is Critical to Trained Immunity. Cell.

[29] Ledford H. (2021). Could mixing COVID vaccines boost immune response?. Nature.

[30] Garg I., Sheikh A.B., Pal S., Shekhar R. (2022). Mix-and-Match COVID-19 Vaccinations (Heterologous Boost): A Review. Infect. Dis. Rep..

[31] Hillus D., Schwarz T., Tober-Lau P., Vanshylla K., Hastor H., Thibeault C., Jentzsch S., Helbig E.T., Lippert L.J., Tscheak P. (2021). Safety, reactogenicity, and immunogenicity of homologous and heterologous prime-boost immunisation with ChAdOx1 nCoV-19 and BNT162b2: A prospective cohort study. Lancet Respir. Med..

[32] Baldolli A., Fournier A., Verdon R., Michon J. (2022). Reactogenicity among health care workers following a BNT162b2 or mRNA-1273 second dose after priming with a ChAdOx1 nCOV-19 vaccine. Clin. Microbiol. Infect..

[33] Junker D., Becker M., Wagner T.R., Kaiser P.D., Maier S., Grimm T.M., Griesbaum J., Marsall P., Gruber J., Traenkle B. (2022). Antibody binding and ACE2 binding inhibition is significantly reduced for both the BA1 and BA2 omicron variants. Clin. Infect. Dis..

[34] Nachtigall I., Bonsignore M., Hohenstein S., Bollmann A., Günther R., Kodde C., Englisch M., Ahmad-Nejad P., Schröder A., Glenz C. (2022). Effect of gender, age and vaccine on reactogenicity and incapacity to work after COVID-19 vaccination: A survey among health care workers. BMC Infect. Dis..

[35] Beatty A.L., Peyser N.D., Butcher X.E., Cocohoba J.M., Lin F., Olgin J.E., Pletcher M.J., Marcus G.M. (2021). Analysis of COVID-19 Vaccine Type and Adverse Effects Following Vaccination. JAMA Netw. Open.

[36] Statistisches Bundesamt Gesundheitspersonal Deutschland. https://www.destatis.de/DE/Themen/Gesellschaft-Umwelt/Gesundheit/Gesundheitspersonal/_inhalt.html.

