# Supplementary material for: Vaccine Side Effects in Health Care Workers after Vaccination against SARS-CoV-2: Data from TüSeRe:exact Study
Source: Viruses. 2022 Dec 25;15(1):65. doi: 10.3390/v15010065 (PMC9864657; doi:10.3390/v15010065)
Supplement: Supplementary file 1 [file viruses-15-00065-s001.zip › viruses-2015365-supplementary.pdf]

Table S1. Generalized linear model across all vaccination instances (i.e. V1, V2 and V3) for local SE according to age groups.

Age	Parameter	p	Odds Ratio	95% Confidence Interval Lower	Upper
18-45 years	BNT vs. AZE	0.276	1.217	0.855	1.733
	MOD vs. AZE	<0.001	2.391	1.456	3.927
	MOD vs. BNT	0.004	1.965	1.239	3.117
	females vs. males	<0.001	1.836	1.316	2.561
	Skin disease	0.019	2.866	1.191	6.898
	Previous COVID-19 infection	0.620	0.810	0.353	1.861
>45 years	BNT vs. AZE	0.190	1.235	0.901	1.684
	MOD vs. AZE	<0.001	2.103	1.441	3.071
	MOD vs. BNT	0.006	1.703	1.163	2.494
	females vs. males	<0.001	1.788	1.316	2.429
	Skin disease	0.060	2.921	0.954	8.943
	Previous COVID-19 infection	0.209	0.612	0.285	1.317

Table S2. Generalized linear model across all vaccination instances (i.e. V1, V2 and V3) for local SE according to age groups.

Age	Parameter	p	Odds Ratio	95% Confidence Interval Lower	Upper
18-45 years	BNT vs. AZE	<0.001	0.196	0.123	0.312
	MOD vs. AZE	0.007	0.485	0.287	0.820
	MOD vs. BNT	<0.001	2.469	1.766	3.454
	females vs. males	0.004	1.563	1.157	2.113
	Skin disease	0.472	1.206	0.724	2.010
	Cardiovascular disease	0.191	1.793	0.748	4.302
>45 years	BNT vs. AZE	<0.001	0.588	0.431	0.804
	MOD vs. AZE	0.598	0.912	0.649	1.282
	MOD vs. BNT	0.006	1.551	1.134	2.121
	females vs. males	<0.001	1.910	1.403	2.600
	Skin disease	0.121	2.167	0.814	5.766
	Cardiovascular disease	0.108	1.539	0.909	2.605

Table S3. Generalized linear model across all vaccination instances (i.e. V1, V2 and V3) for local SE in males and females.

Gender	Parameter	p	Odds Ratio	95% Confidence Interval Lower	Upper
male	BNT vs. AZE	0.005	1.899	1.211	2.979
	MOD vs. AZE	<0.001	3.321	1.827	6.036
	MOD vs. BNT	0.043	1.749	1.019	3.002
	Age 18-45 vs. Age >45	0.010	1.736	1.141	2.640
	Skin disease	0.014	5.417	1.416	20.725
	Previous COVID-19 infection	0.171	0.507	0.191	1.343
female	BNT vs. AZE	0.629	0.936	0.716	1.224
	MOD vs. AZE	<0.001	1.952	1.386	2.748
	MOD vs. BNT	<0.001	1.827	1.315	2.538
	Age 18-45 vs. Age >45	<0.001	1.866	1.425	2.444
	Skin disease	0.014	2.475	1.200	5.106
	Previous COVID-19 infection	0.358	0.748	0.402	1.390

Table S4. Generalized linear model across all vaccination instances (i.e. V1, V2 and V3) for systemic SE in males and females.

	Parameter	p	Odds Ratio	95% Confidence Interval Lower	Upper
male	BNT vs. AZE	<0.001	0.408	0.256	0.651
	MOD vs. AZE	0.294	0.745	0.429	1.292
	MOD vs. BNT	0.005	1.824	1.194	2.786
	Age 18-45 vs. Age >45	0.007	1.386	1.092	1.759
	Skin disease	0.105	2.045	0.860	4.864
	Cardiovascular disease	0.133	0.582	0.847	3.487
female	BNT vs. AZE	<0.001	0.402	0.303	0.533
	MOD vs. AZE	0.183	0.799	0.575	1.112
	MOD vs. BNT	<0.001	1.989	1.520	2.603
	Age 18-45 vs. Age >45	0.007	1.386	1.092	1.759
	Skin disease	0.195	1.476	0.819	2.661
	Cardiovascular disease	0.122	1.532	0.892	2.630
